# Fluoride‐Induced Corrosion of Stainless Steel: A Case Study for its Application as Proton Exchange Membrane Water Electrolysis Bipolar Plate Material

**DOI:** 10.1002/cssc.202501561

**Published:** 2025-09-22

**Authors:** Lena Fiedler, Darius Hoffmeister, Tien‐Ching Ma, Julian Schwarz, Ferdinand Günther, Thomas Przybilla, Erdmann Spiecker, Simon Thiele, Dominik Dworschak, Karl J. J. Mayrhofer, Andreas Hutzler

**Affiliations:** ^1^ Helmholtz Institute Erlangen‐Nürnberg for Renewable Energy (IET 2) Forschungszentrum Jülich GmbH Cauerstraße 1 91058 Erlangen Germany; ^2^ Department of Chemical and Biological Engineering Friedrich‐Alexander‐Universität Erlangen‐Nürnberg Egerlandstraße 3 91058 Erlangen Germany; ^3^ Electron Devices (LEB) Friedrich‐Alexander‐Universität Erlangen‐Nürnberg Cauerstraße 6 91058 Erlangen Germany; ^4^ Institute of Micro‐ and Nanostructure Research & Center for Nanoanalysis and Electron Microscopy (CENEM) Friedrich‐Alexander‐Universität Erlangen‐Nürnberg Cauerstraße 3 91058 Erlangen Germany

**Keywords:** 316L Dissolution, Hydrogen Production, Pitting Corrosion, Stability, Water Splitting

## Abstract

Stainless steel is a promising material for bipolar plates (BPP) in proton exchange membrane water electrolysis (PEMWE) that could drastically reduce stack costs. However, dissolution of Fe from stainless steel BPP might trigger membrane degradation, which releases fluoride. Fluoride in turn could accelerate stainless steel corrosion. Therefore, the influence of fluoride contamination (namely 0, 1, 5, and 20 ppm in 0.5 mM H_2_SO_4_) on the dissolution stability of stainless steel (316L) is investigated utilizing a scanning flow cell coupled on‐line to an inductively coupled plasma mass spectrometer (SFC‐ICP‐MS). Fluoride enhances the dissolution exponentially, resulting in enhanced dissolution efficiencies with increased fluoride concentration reaching ≈50% at 20 ppm. Complementary micro and nanostructure analysis (laser profilometry, scanning electron microscopy, and scanning transmission electron microscopy with energy‐dispersive X‐ray spectroscopy) reveals pitting corrosion, whose severity and occurrence appear highly increased with higher fluoride concentration. The results suggest that fluoride impurities in combination with exposed stainless steel, e.g., due to coating imperfections, should be avoided in PEMWE application, as accumulation of impurities of both might lead to a self‐accelerating degradation process.

## Graphical abstract

In this study, the influence of fluoride on the dissolution stability of stainless steel is investigated on‐line. Fluoride significantly enhances dissolution with increased pitting corrosion. The amount and severity of pits increase with higher fluoride concentration. The results shed light on the possibility of a dangerously self‐accelerating degradation mechanism of electrolyzers by stainless steel dissolution.

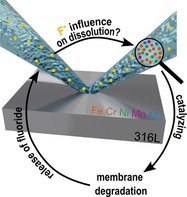

## 1 Introduction

For the defossilization of our society, green hydrogen—hydrogen produced from water by electrolysis using renewable energies—is urgently needed. However, the high costs render the technology poorly competitive with fossil fuel‐based hydrogen, for instance, as a reactant in industry, making a cost reduction indispensable.^[^ ^1^, ^2^ ^]^ When coupled with renewable energies, proton exchange membrane water electrolysis (PEMWE) is the most beneficial technology due to its fast system response upon fluctuating energy input and high achievable current densities.^[^ ^3^, ^4^ ^]^ Besides expensive noble metal‐based catalysts,^[^ ^5^, ^6^ ^]^ the bipolar plates (BPP) are a detrimental factor of PEMWE costs.^[^ ^7^ ^]^

BPPs separate the individual cells of a proton exchange membrane (PEM) water electrolyzer, transport water and gases, and ensure efficient conduction of heat and current.^[^ ^8^ ^]^ Especially for the anode side of the BPP, operational conditions were long assumed to be quite harsh, with a low pH value and high potential, whereas the latest research hints at lower potentials and higher pH values compared to conditions at the catalyst layer.^[^ ^9^ ^]^ The state‐of‐the‐art BPP material titanium is usually coated with noble metals such as gold or platinum, as a corrosion‐resistant material with a high thermal and electrical conductivity, as well as a low interfacial contact resistance (ICR) to the adjacent porous transport layer (PTL) is required.^[^ ^10^, ^11^ ^]^ Therefore, BPPs are the cause for more than 50% of the stack costs and require significant cost reduction.^[^ ^7^ ^]^ Many alternative materials are investigated, with stainless steel being the up‐and‐coming material, whose applicability, bare and coated, was tested by many research groups with promising results.^[^ ^8^, ^12^, ^13^, ^14^, ^15^, ^16^, ^17^, ^18^, ^19^, ^20^, ^21^, ^22^, ^23^ ^]^ The main advantages are the lower material costs and the more facile processing compared to Ti.^[^ ^10^ ^]^ Stiber et al.^[^ ^16^, ^17^ ^]^ achieved outstanding results using a Ti‐ and Ti/Nb‐coated stainless steel BPP and PTL on single cell level. Our previous studies showed that stainless steel dissolves under conditions typically assumed at the BPP.^[^ ^14^, ^23^ ^]^ Fe, the primary alloy component of stainless steel, is known to degrade electrolyzers already at low concentrations in the water feed.^[^ ^24^, ^25^ ^]^ Besides decreasing the conductivity of the membrane, as known from *ex situ* tests,^[^ ^26^, ^27^, ^28^ ^]^ Fe acts as a catalyst‐like agent in membrane degradation. As the commonly used membrane in PEMWE is Nafion, a perfluorinated polymer (Figure S1, Supporting Information), its chemical degradation can lead to fluoride release into the water feed.^[^ ^29^, ^30^ ^]^ Dissolved Fe^3+^ from the anode migrates through the membrane to the cathode side and is reduced to Fe^2+^. Fe^2+^ can catalyze a Fenton‐based reaction that releases hydroxy radicals (Equation 1).^[^ ^29^, ^31^, ^32^, ^33^ ^]^ Moreover, the direct reaction of Fe^3+^ can cause Fe^2+^ and hydroperoxyl radical formation (Equation 2).^[^ ^29^ ^]^ The required hydrogen peroxide stems from the reduction of crossover oxygen at the platinum cathode catalyst.^[^ ^34^ ^]^ The formed radicals can attack the Nafion polymer at the perfluorinated backbone and perfluorosulfonic side chains or at weak end groups such as —COOH, leading to fluoride release (Equation 3).^[^ ^31^, ^35^ ^]^ Even though the Fe source is at the anode side, membrane thinning of PEMWE was observed mainly at the cathode side.^[^ ^25^, ^36^, ^37^ ^]^ However, it may also occur at the anode side,^[^ ^38^ ^]^ and fluoride contaminations were found in the anode water feed.^[^ ^30^, ^34^, ^39^ ^]^ Moreover, it is suspected that other metal cationic impurities can act as catalysts for the reactions.^[^ ^30^ ^]^ Therefore, the dissolution of Cr, Ni, Mn, and Mo, the other main alloy elements of stainless steel, might harm the system in similar ways.

(1) $${\text{Fe}}^{\text{2+}}\;\text{+}\;H_{2}O_{2}\;\text{+}\;H^{+}\to {\text{Fe}}^{\text{3+}}\;\text{+}\;{\text{HO}}^{•}\;\text{+}\;H_{2}O$$

(2) $${\text{Fe}}^{\text{3+}}\;\text{+}\;H_{2}O_{2}\to {\text{Fe}}^{\text{2+}}\;\text{+}\;{{\text{HO}}_{2}}^{•}\;\text{+}\;{\text{ H}}^{+}$$

(3) $${\left({\mathrm{CF}}_{2}\right)}_{j}\text{–COOH}\;\text{+}\;{\text{2 HO}}^{•}\to {\left({\text{CF}}_{2}\right)}_{\text{j‐1}}\text{–COOH}\;\text{+}\;{\text{CO}}_{2}\;\text{+}\;\text{2 HF}$$

Fuel cell research has shown that significant concentrations of fluoride ions increase stainless steel corrosion.^[^ ^40^, ^41^, ^42^, ^43^, ^44^ ^]^ While Laedre et al.^[^ ^44^ ^]^ did not observe significant differences after adding 2 ppm of fluoride to sulfuric acid, Xuan et al.^[^ ^41^ ^]^ observed an increase in corrosion current and a decrease in the corrosion potential. Different testing conditions, such as temperature and acid concentration, are likely to cause this discrepancy. Even though fluoride is often added for *ex situ* BPP testing—used concentrations differ from 0.1,^[^ ^12^, ^20^ ^]^ over 2,^[^ ^18^ ^]^ and up to 5 ppm^[^ ^13^ ^]^ fluoride—to the best of our knowledge, a systematic study investigating the influence of fluoride on the corrosion of 316L for PEMWE application is still missing despite knowledge of its effect is essential. As outlined above, stainless steel dissolution can trigger fluoride release by membrane degradation. If fluoride enhances stainless steel dissolution, triggering further membrane degradation, it may induce a self‐accelerating mechanism that tremendously degrades the electrolyzer. Besides lower efficiency, membrane thinning can result in local hotspot formation and higher gas crossover rates, thus posing significant safety issues.^[^ ^31^, ^37^ ^]^

Consequently, we investigate the influence of fluoride on the dissolution stability of stainless steel (type 316L) utilizing our previously introduced scanning flow cell setup coupled on‐line to an inductively coupled plasma mass spectrometer (SFC‐ICP‐MS).^[^ ^14^ ^]^ We mimic conditions at the anode BPP by adding different amounts of fluoride (0, 1, 5, and 20 ppm with potassium fluoride as fluoride source) to highly diluted sulfuric acid (0.5 mM). The fluoride concentrations used cover the range typically applied in *ex situ* BPP testing for PEMWE applications and additionally include a higher concentration to further explore the trends observed at elevated fluoride levels, similar to studies in the fuel cell field.^[^ ^8^, ^12^, ^13^, ^15^, ^18^, ^20^, ^40^, ^41^, ^42^ ^]^ Complementary laser profilometry, scanning electron microscopy (SEM) imaging, and scanning transmission electron microscopy with energy‐dispersive X‐ray spectroscopy (STEM–EDXS) analysis provide further insights into the dissolution mechanisms observed.

## 2 Results and Discussion

**Figure 1:**
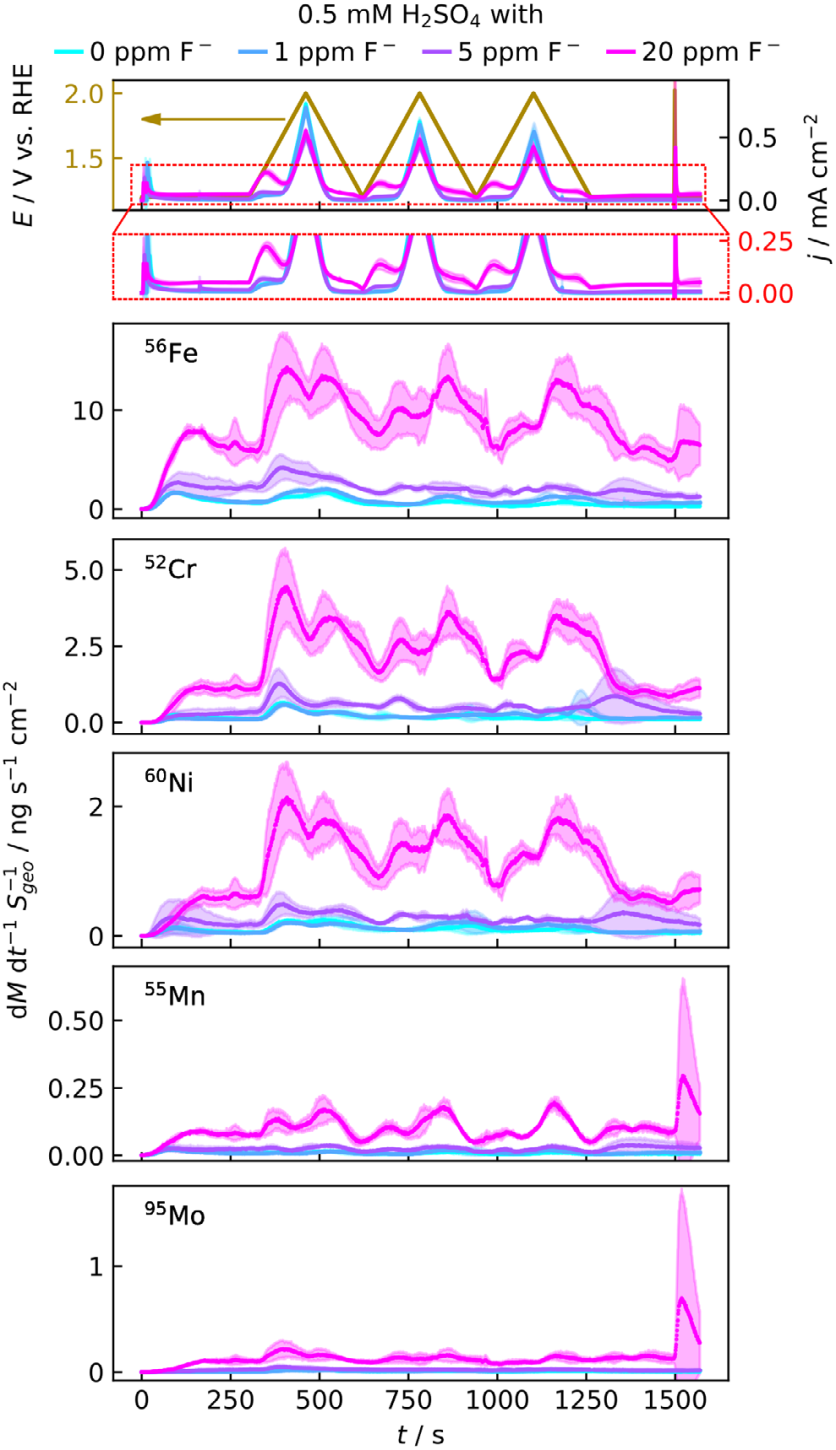
Influence of fluoride on 316L dissolution. Potential profile (beige) and current density, zoom‐in into current density curve, and dissolution rates of Fe, Cr, Ni, Mn, and Mo (from top to bottom) at four different fluoride concentrations (0 ppm – cyan, 1 ppm – blue, 5 ppm – purple, and 20 ppm – magenta)—average of three measurements per fluoride concentration on different spots with standard deviation. Individual measurements are displayed in Figure S3, Supporting Information.

**Figure** **1** displays the dissolution profile of 316L per fluoride concentration during cyclic voltammetry (CV) measurements between 1.2 and 2 V with a previous and subsequent hold at 1.2 V at a stage temperature *T*_stage_ = 60 °C. When contacting the SFC to 316L during the initial 1.2 V hold, a current spike is observed, accompanied by increasing dissolution rates of all elements. While the differences in the dissolution profiles are minor for 0 and 1 ppm fluoride, the dissolution rates increase for 5 and particularly 20 ppm fluoride. The dissolution rates decrease after peaking, especially at lower fluoride concentrations, but do not return to the baseline during the hold.

The current density rises upon potential increase, followed by an increase in dissolution rate for all elements at all fluoride concentrations. For 20 ppm, a distinct current density peak evolves with the maximum at 1.44 ± 0.02 V. Such a peak partly evolves for 5 ppm fluoride, while a plateau is mainly observed for 0 and 1 ppm. We assume that the peak is caused by a weaker passivation of 316L due to fluoride, which is also observed in literature.^[^ ^41^ ^]^ This is in accordance with the significantly higher dissolution rates at increased fluoride concentrations. Upon further potential increase, a second steep current density increase evolves, which we assign to the oxygen evolution reaction (OER). Many alloy elements of 316L are known OER catalysts, even though they are applied in alkaline water electrolysis for stability reasons.^[^ ^45^, ^46^ ^]^ Across all electrolytes, the dissolution rate decreases before a second peak, appearing with a maximum in the backward scan, emerges during the first CV cycle.

**Figure 2:**
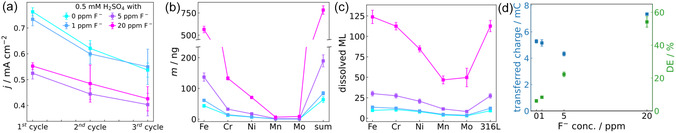
Analysis of the SFC–ICP–MS measurements of 316L displayed in Figure 1. Average values of 3 measurements per fluoride concentration on different spots with standard deviation. a) Maximum current density during the three CV cycles, b) dissolved mass per element and sum of all, c) respective dissolved ML per element and for 316L if the dissolution would have occurred as expected from the elemental composition, and d) transferred charge and DE during the whole measurement.

Upon cycling, the transferred charge per cycle and maximum current density at the vertex potential decrease for all fluoride concentrations (**Figure** **2**a and Table S2, Supporting Information). The maximum current density is higher for 0 and 1 ppm than for 5 and 20 ppm of added fluoride, which hints at a decreased OER activity/efficiency. Ma et al.^[^ ^47^ ^]^ also suggested a decreased OER efficiency by fluoride, however, for IrO_2_—Ta_2_O_5_‐coated Ti anodes. For the dissolution profiles, the situation is more complex. Generally, the maximum dissolution rates decrease with cycling (Table S3, Supporting Information). Combined with the decreasing current density, this indicates passivation. However, the dissolution rate differences of the individual measurement per fluoride concentration increase with cycling, and the deviation is higher with increased fluoride concentration (Figure S3, Supporting Information). A closer look reveals sudden, unexpected increases in the dissolution rate, often accompanied by a transient in the current signal, as elucidated in the Supporting Information. One possible explanation is a breakdown of the formed oxide layer, e.g., due to the flow or OER. However, such transients are also known to be caused by metastable pits and pit nucleation.^[^ ^48^, ^49^ ^]^

The dissolution is never inhibited in all measurements—during the final 1.2 V hold, the current density rises, and the dissolution rates are above the baseline level. Both increase with higher fluoride concentration, again, showing the reduced passivation and dissolution stability of 316L by fluoride. Figure 2b displays the dissolved mass per element and the total sum over the entire measurement time. The dissolution‐enhancing effect is also visible in the cumulative dissolved mass, which increases exponentially with increasing fluoride concentration (Figure 2b). From the dissolved mass, we calculated its equivalents in dissolved monolayer (ML) (Figure 2c, for calculation details refer to the Supporting Information). For all fluoride concentrations, Cr dissolves at a rate as expected from 316L's elemental composition, while Mn and Mo dissolve below their stoichiometric composition. Fe and Ni dissolve at a rate as expected for no fluoride, whereas Fe dissolves preferentially with increasing concentration, while Ni dissolution is slightly suppressed. A possible explanation for the differences in the general element dissolution and trends with fluoride concentration is the different ability of the metals to form metal–fluoride compounds.^[^ ^50^ ^]^ For example, it has been reported that Ni preferentially reacts and deposits as NiF_2_ on 904 L compared to the other alloy elements; however, this study was conducted in significantly higher concentrated pure HF.^[^ ^51^ ^]^ By X‐ray photoelectron spectroscopy (XPS) depth profiling Yang et al.^[^ ^42^ ^]^ reported a preferred Fe dissolution compared to Cr from 316L after 5 h polarization of 316L at −0.1 V_SCE_ (0.01 mM H_2_SO_4_ with 0, 5.7, 11.4, 19, and 95 ppm fluoride), and the Cr/Fe‐ratio decreased with increased fluoride concentration. In a related study,^[^ ^40^ ^]^ they reported that after polarization to 0.6 V_SCE_ an Fe‐rich oxide layer formed regardless of the fluoride concentration. Xuan et al.^[^ ^41^ ^]^ observed a decrease in the Ni content, while the Cr and Fe content were comparable to the pristine state of 304 after electrochemical measurement (0.6 V_SCE_ for 5 h in 0.01 mM H_2_SO_4_ with 0, 2, 20, and 200 ppm fluoride) by EDXS. This was observed for all fluoride concentrations. In the last three studies mentioned, the dissolution of alloy components into the electrolyte was not investigated. As these studies focus on fuel cell applications, the applied potentials are considerably lower than those used in our work. Moreover, they employed potentiostatic holds, whereas we subjected the samples to potential cycling. Both differences and a different acid concentration can explain the observed divergence. Because 316L is a multicomponent alloy, its dissolution behavior will reflect a complex interplay among all elements.

The highly dissolution‐enhancing effect of fluoride on 316L is visible in Figure 2d. Even though the transferred charge decreases initially with increasing fluoride concentration, the dissolution efficiency (DE)—the percentage of current consumed for dissolution—increases (for calculation details, refer to Supporting Information), reaching 54.2 ± 3.3% for 20 ppm fluoride. This shows that most of the transferred charge is consumed for dissolution, and other competing reactions, such as OER or oxide layer formation, are suppressed. Figure S5, Supporting Information, displays the DE split into different current regimes during cycling. Here, it becomes apparent that the DE is constantly high for 20 ppm, while it changes for the other concentrations, showing again the high amount of dissolution regardless of the potential applied.

**Figure 3:**
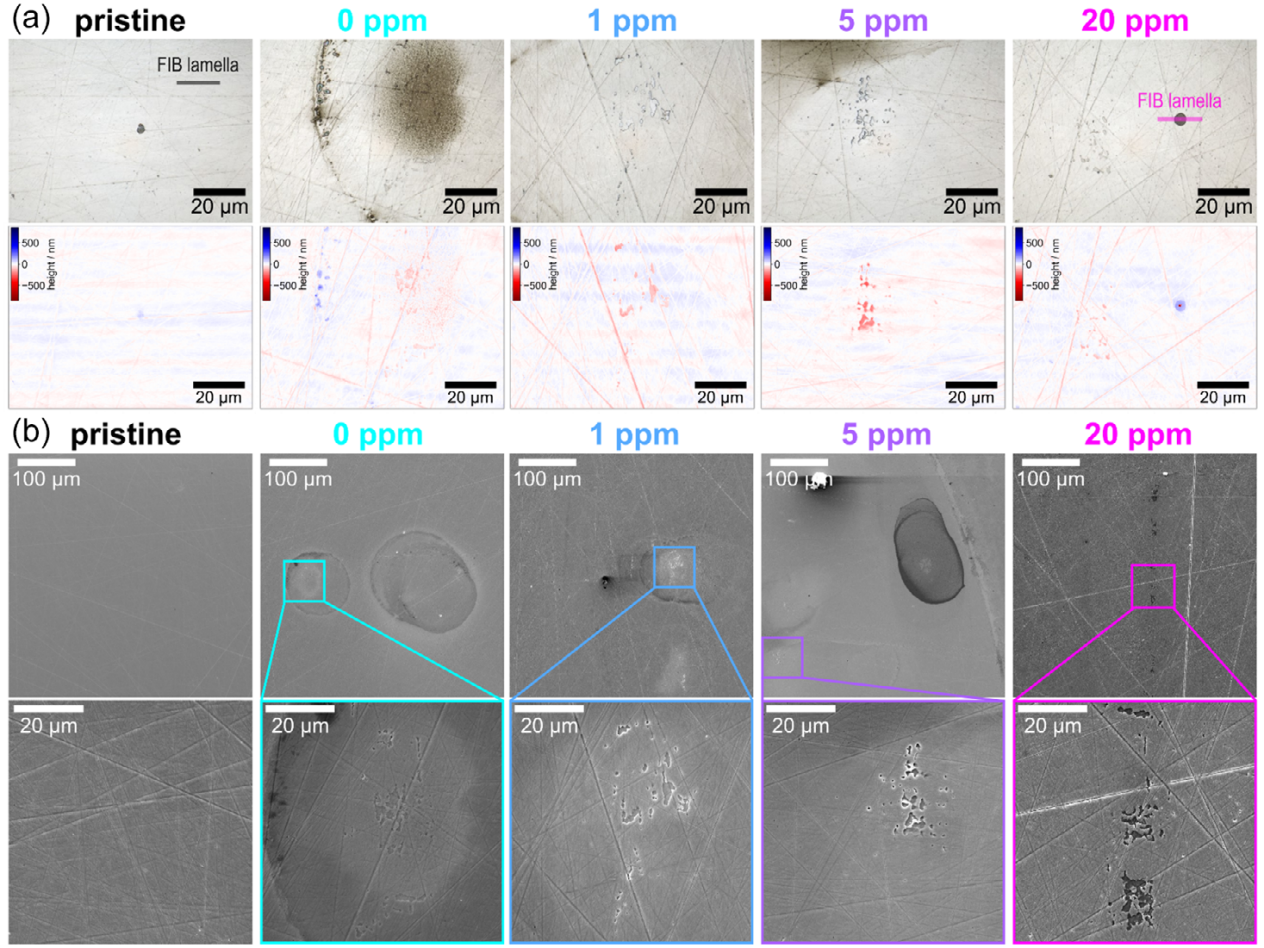
Correlative light microscopy, laser profilometry, and SEM analysis of 316L in its pristine state and after SFC–ICP–MS measurements in 0.5 mM H_2_SO_4_ with different fluoride concentrations. a) Laser optical micrograph and corresponding laser profilometry height map. b) Exemplary surface SEM images – additional SEM images can be found in Figures S5 and S6, Supporting Information. All images were contrast enhanced to improve visibility.

After measurement, corrosion phenomena are already visible with light microscopy, and laser profilometry measurements reveal pitting corrosion, with one symptom being accumulations of low‐recessed areas (**Figure** **3**a). Further structural analysis by SEM shows the increased severity of pitting with increased fluoride concentration. On all samples, macroscopic signs of corrosion are visible; however, nonuniformly distributed over the spot area (Figure S6, Supporting Information). As an O‐ring is used as a sealant, crevice corrosion can also occur.^[^ ^52^ ^]^ The high‐magnification SEM images reveal different corrosion phenomena depending on the fluoride concentration (Figure 3 and S7, Supporting Information). While few pitting was detected for 0 ppm fluoride, the number apparently increases with increasing fluoride concentration. Especially after the measurement with 20 ppm fluoride, multiple areas with pitting corrosion are visible, which agrees with the high observed dissolution during the measurements. The depth of these pits is in the low hundred‐nm range as measured by laser profilometry (Figure 3a). Besides, laser profilometry measurements indicate deposits with an apparent recess in the center (Figure 3a‐20 ppm and Figure S8a, Supporting Information), and such sites appear noticeable in SEM images (Figure S8b, Supporting Information), however, without the recess. To further analyze this, a TEM lamella was prepared using focused ion beam (FIB) preparation at one of the deposits with apparent recess after measurement with 20 ppm fluoride and a pristine area. STEM–EDXS analysis (Figure S9, Supporting Information) reveals that the area with suspicious corrosion is an inclusion that mainly consists of Mn, Al, and Mg. We also could not verify the apparent pit by STEM–EDXS (possible reasons are elucidated in the Supporting Information) despite such inclusions being generally known pitting initiation sites for stainless steel corrosion.^[^ ^53^, ^54^, ^55^, ^56^, ^57^ ^]^ In sum, the microscopic analysis clearly supports the increased corrosion by fluoride and pitting of 316L.

Pitting corrosion of stainless steel in the presence of fluoride has also been reported by other researchers for type 316L,^[^ ^40^ ^]^ whereas no such effect was observed for type 304.^[^ ^41^ ^]^ Apart from the different steel grades, the main distinction between these studies lies in the testing temperature (70 °C vs. ambient temperature). Since temperature is a known factor that enhances stainless steel corrosion for both grades,^[^ ^23^, ^58^, ^59^ ^]^ we assume that this difference can account for the occurrence or absence of pitting. In this study, the application of an elevated temperature (*T*_stage_ = 60 °C) together with higher potentials compared to both other studies provides a plausible explanation for the pitting observed here. The observed occurrence of pitting corrosion can explain the dissolution profile differences detected between the individual measurements. As mentioned above and discussed in the Supporting Information, pitting initiation can cause the observed current transients and unexpected increases in the dissolution rate. Supposing that initiated pits continue to propagate—which is particularly likely at higher fluoride concentrations—the electrochemical conditions inside the pits differ from those on the rest of the surface, with locally lower pH and potential.^[^ ^48^, ^49^ ^]^ Therefore, the corrosion behavior inside the pit might vary, resulting in different dissolution rate courses depending on the pit initiation and propagation, which could be different for different measurement spots, for example, depending on the amount of inclusions.^[^ ^53^, ^54^ ^]^ As the applied potentials are above pitting potentials reported for stainless steel in fluoride presence,^[^ ^40^ ^]^ we assume that pitting might not be inhibited again if propagated. As the sampling area of an SFC is comparably small (*A* = 4.16 mm^2^), such local effects are apparent in the overall signal, whereas for larger sampling areas they can be obscured by the general larger current signals or, if measured, on‐line dissolution rates.

If used as BPP, 316L will require coatings as already widely tested in literature,^[^ ^13^, ^15^, ^16^, ^17^, ^18^, ^19^, ^20^, ^21^, ^22^ ^]^ on the one hand to prevent contamination and on the other hand to reduce the ICR to the PTL. Our results show the significantly dissolution‐enhancing effect of fluoride on the dissolution of 316L, and the question arises if that could have detrimental effects on cell level whenever uncoated stainless steel is present, e.g., due to coating imperfections. Reported fluoride emission rates (FER) at cell level differ. Commonly, reported values are higher for the cathode than for the anode water outlet.^[^ ^34^, ^39^, ^60^, ^61^, ^62^ ^]^ The highest reported FER are ≈3.5^[^ ^62^ ^]^ and 0.83 ± 0.02 μg cm^−2^ h^−1^^[^ ^60^ ^]^ for the cathode and anode side, respectively. Accumulation of 1 ppm fluoride with these FER occurs if the water flow rate is ≤0.06 mL min^−1^ cm^−2^, which is significantly lower than the commonly used rate of ≥2 mL min^−1^ cm^−2^^[^ ^63^, ^64^ ^]^ (for calculation, see Supporting Information). Of course, this only holds true if the recirculating water is adequately cleaned by ion exchangers to avoid accumulation over operation time. Moreover, as FER are measured *ex situ*, the concentration inside the cell, especially locally, might be significantly higher. In addition, different operations, e.g., start‐stop‐cycling, or the presence of (cationic) impurities due to degradation, might lead to higher FER and thereby accelerated dissolution.

## 3 Conclusion

In summary, we investigated the influence of fluoride on the dissolution of 316L in 0.5 mM H_2_SO_4_. The results clearly show the lower dissolution stability of 316L with increasing fluoride concentration, triggering pitting corrosion. Therefore, whenever applied as BPP material in PEM electrolyzers, the dissolution of stainless steel should be prevented by defect‐free coatings that fully inhibit the dissolution of the base material. Otherwise, dissolution of cations, inducing membrane degradation with fluoride release, which triggers further stainless steel dissolution, is a potential risk. If appropriately coated, stainless steel could be an ideal base material for BPPs that will contribute to reducing the costs of green hydrogen produced with PEMWE. The dissolution stability of possible coating materials for PEMWE BPPs and the influence of fluoride on the open‐circuit dissolution of unprotected stainless steel, e.g., for balance of plants components,^[^ ^23^ ^]^ including single‐cell testing, is subject to future investigations.

## 4 Experimental Section

4.1

4.1.1

##### Materials

316L (*d* = 1 mm, purchased from Hans‐Erich Gemmel & Co GmbH) was ground (SiC, from P240 to P4000, Struers) and polished with 3 μm diamond suspension (Mol B3, Struers) with a semi‐automated polishing machine (LaboPol‐30 with LaboForce 100, Struers). The samples were cleaned with isopropanol and deionized (DI) water before and after ultrasonication in DI water (2 × 10 min). Until measurement, the samples were stored in ambient air for 10 and 11 days, respectively. The complete formation of the oxide film and thereby comparable surface between two measurement days was proven by optical microspectroscopy (section S3, Supporting Information).

For the reflectance measurements, the sample was additionally polished with ¼ μm diamond suspension (Nap ¼, Struers) and 0.04 μm colloidal silica suspension (OP‐U, Struers). Afterward, the sample was cleaned with soap solution followed by the cleaning procedure described above.

##### Electrochemical Measurements

All electrochemical measurements were performed with a scanning flow cell coupled on‐line to an inductively coupled plasma mass spectrometer (SFC‐ICP‐MS). A detailed description of the setup can be found elsewhere.^[^ ^14^, ^65^ ^]^ In short, the electrolyte was constantly pumped through the SFC's V‐shaped channel, which had an opening at the channel's vertex to contact the SFC to the sample. The sample was placed on a temperature‐controlled stage and heated to *T*_stage_ = 60 °C. A glassy carbon rod (Ø = 1.6 mm, HTW Hochtemperatur–Werkstoffe) used as CE was placed in the inlet, and a leak‐free Ag|AgCl‐reference electrode (LF–1.6–48, Ø = 1.6 mm, Innovative Instruments, Inc.) placed in the outlet. 0.5 mM Ar‐saturated H_2_SO_4_ (diluted from H_2_SO_4_ (Ultrex II, J.T.Baker) with ultrapure water (18.2 MΩ cm^−1^)) with 0, 1, 5, and 20 ppm fluoride (by addition of potassium fluoride (99.99%, Thermo Scientific), ppm refers to the fluoride content) was used as electrolyte. As the electrolytes contained fluoride, the silicone‐based O‐ring conventionally used at the SFC‐opening was chemically unstable. Instead, an FPM O‐ring (Ø_i.d_ = 2.3 mm, cross section = 0.8 mm, Arcus) was placed below the SFC opening to seal the sample's active area by pressing the SFC with a force of 7 N controlled by a force sensor (KD45 10 N, ME‐Meßsysteme) on the O ring. The active area (*A *= 4.16 mm^2^) was calculated based on the inner diameter Ø_i.d_ of the O‐ring. All reported potentials were shifted to the reversible hydrogen electrode (RHE). Therefore, the open circuit potential of the RE was measured versus a Pt wire (Ø = 0.5 mm, 99.99%, MaTeck) in hydrogen‐saturated electrolyte. With a potentiostat (Reference 620, Gamry Instruments), the following electrochemical protocol was used: Hold at 1.2 V for 300 s, during which the SFC was contacted to the sample, followed by three CV cycles from 1.2 to 2 V (*v *= 5 mV s^−1^) and a final 300 s hold at 1.2 V. Three measurements on three different sample spots were performed per electrolyte.

The downstream electrolyte was constantly fed into an ICP–MS (7900, Agilent Technologies) operated in low matrix mode with a He‐collision cell (*V*_He_ = 4.3 mL min^−1^) in time‐resolved analysis. Before entering, the electrolyte was mixed with an internal standard solution (^72^Ge for ^52^Cr, ^55^Mn, ^56^Fe, ^60^Ni, and ^95^Mo in 5% HNO_3_ (CarlRoth, Ultrapur) prepared from Certipur ICP‐MS standard, Merck). Calibration of the ICP–MS with standard solutions (Certipur ICP‐MS standard, Merck) and measurement of the flow rate from the SFC to the ICP–MS was performed daily for each electrolyte.

Directly after measurement, the measurement spot was carefully flushed with water and dried. Before plotting, the dissolution profiles were baseline corrected by subtracting the minimum value measured per element and measurement from the dissolution rate. For all data handling, evaluation, and visualization an SQL and Python‐based data management tool was used.^[^ ^66^ ^]^

##### Optical Microspectroscopy

Potential oxide growth on a newly polished 316L sample was investigated over a period of 11 days via reflectance microspectroscopy, as described in detail in previous works.^[^ ^67^, ^68^, ^69^, ^70^ ^]^ Reflectance spectra were recorded with a 10×/NA 0.25 objective lens coupled to a Horiba iHR 320 spectrometer equipped with a 1200 g mm^−1^ diffraction grating and a Horiba Synapse CCD detector. The refractive indices for Air,^[^ ^71^ ^]^ Si,^[^ ^72^ ^]^ 316L,^[^ ^73^ ^]^ and Cr_2_O_3_^[^ ^74^ ^]^ were taken from literature.

##### Laser Profilometry

Laser profilometry measurements and microscopic images were acquired with a laser scanning confocal microscope (VK–X1000, Keyence) of the measurement spots after SFC‐ICP–MS measurements and one pristine area. The obtained height profiles were background corrected.

##### TEM‐Lamellae Preparation and STEM–EDXS

For TEM characterization, thin cross‐section lamellae were extracted using the FIB lift‐out method (Helios NanoLab 660 DualBeam FIB‐SEM, Thermo Fisher, USA). The lamellae were milled down with the Ga^+^ beam to a thickness of ≈90 nm to achieve electron transparency by gradually lowering the acceleration voltage from 30 to 2 kV.

High‐angle annular dark field scanning transmission electron microscopy (HAADF‐STEM) and STEM‐EDXS were performed using a Talos F200i (Thermo Fisher Scientific) operated at an acceleration voltage of 200 kV. A probe current of roughly 40 pA and a convergence angle of 10.5 mrad were adjusted.

## Supporting Information

The authors have cited additional references within the Supporting Information.^[^ ^75^, ^76^, ^77^, ^78^, ^79^, ^80^ ^]^

## Conflict of Interest

The authors declare no conflict of interest.

## Supporting information

Supplementary Material

## Data Availability

The data that supports the findings of this study are available from the corresponding author upon reasonable request.
