# Supplementary material for: Fluoride‐Induced Corrosion of Stainless Steel: A Case Study for its Application as Proton Exchange Membrane Water Electrolysis Bipolar Plate Material
Source: ChemSusChem. 2025 Sep 22;18(21):e202501561. doi: 10.1002/cssc.202501561 (PMC12584964; doi:10.1002/cssc.202501561)
Supplement: Supplementary file 1 — Supplementary Material [file CSSC-18-e202501561-s001.pdf]

Supporting Information

Fluoride-Induced Corrosion of Stainless Steel: A Case Study for its Application as PEMWE Bipolar Plate Material

Lena Fiedler*,[a,b] Darius Hoffmeister,[a,b] Tien-Ching Ma,[a,b] Julian Schwarz,[c] Ferdinand Günther,[d] Thomas Przybilla,[d] Erdmann Spiecker,[d] Simon Thiele,[a,b] Dominik Dworschak,[a] Karl J. J. Mayrhofer,[a,b] and Andreas Hutzler*[a]

[a] L. Fiedler, D. Hoffmeister, T.-C. Ma, Prof. Dr. S. Thiele, Dr. D. Dworschak, Prof. Dr. K. J. J. Mayrhofer, Dr. A. Hutzler
Helmholtz Institute Erlangen-Nürnberg for Renewable Energy (IET 2)
Forschungszentrum Jülich GmbH
Cauerstraße 1, 91058 Erlangen (Germany)
E-mail: l.fiedler@fz-juelich.de, a.hutzler@fz-juelich.de

[b] L. Fiedler, D. Hoffmeister, T.-C. Ma, Prof. Dr. S. Thiele, Prof. Dr. K. J. J. Mayrhofer
Department of Chemical and Biological Engineering
Friedrich-Alexander-Universität Erlangen-Nürnberg
Egerlandstraße 3, 91058 Erlangen (Germany)

[c] J. Schwarz
Electron Devices (LEB)
Friedrich-Alexander-Universität Erlangen-Nürnberg
Cauerstraße 6, 91058 Erlangen (Germany)

[d] F. Günther, Dr. T. Przybilla, Prof. Dr. E. Spiecker
Institute of Micro- and Nanostructure Research & Center for Nanoanalysis and Electron Microscopy (CENEM)
Friedrich-Alexander-Universität Erlangen-Nürnberg
Cauerstraße 3, 91058 Erlangen (Germany)

1 Chemical structure of Nafion

Figure S1 displays the chemical structure of Nafion, a perfluorinated polymer, that is commonly used as membrane for PEM electrolyzers.[1-2]

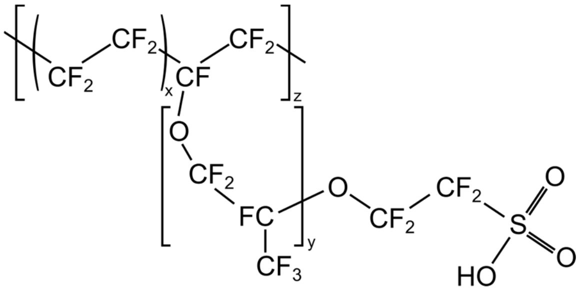

Figure S1. Chemical structure of Nafion.

2 Calculation of dissolved monolayers and dissolution efficiency

To give the reader a better understanding of the dissolution during SFC-ICP-MS measurements, we calculated the theoretical equivalent of the dissolved mass in dissolved monolayers (ML). This was done in the same manner as in our previous publications.[3-4] In short, a face-centered cubic crystal structure of 316L with the lattice constant a = 0.359 nm[5-6] and the elemental composition of 316L given in Table S1 were used to calculate the amount of metal per element and 316L if one ML of the most densely packed plane ((111) plane) dissolves. Dividing the dissolved mass during the SFC-ICP-MS measurements by the obtained values approximates the dissolved MLs.

Table S1. Composition of 316L in wt% used for all calculations based on the average values with Fe at balance of the theoretical composition of 316L[7], which was verified by STEM-EDXS measurements (spectrum image Figure S9a, region of interest in the yellow rectangle).

	Fe	Cr	Ni	Mn	Mo	O	C
Used composition	65.5	17	12	2	2.5	-	-
STEM EDXS	67.2 ± 3.5	16.2 ± 2.4	9.9 ± 1.6	1.4 ± 0.3	4.9 ± 0.8	0.5 ± 0.1	0.2 ± 0.1

To calculate the dissolution efficiency (DE), we integrated the current measured during the SFC-ICP-MS measurements to obtain the transferred charge. In general, the current is a combination of different electrochemical processes occurring on the sample surface, such as oxidation resulting in the dissolution or formation of an oxide layer on the sample's surface, oxygen evolution reaction at elevated potential, or double layer-charging. The DE describes the percentage of charge transferred that is consumed for the dissolution of 316L calculated from the dissolution data as described previously.[3-4, 8] Shortly, based on the Pourbaix-Diagrams, we assumed equations (1) – (5) as oxidation reactions, enabling the calculation of the DE with equation (6).

$$2\,Fe + 3\,H_2O \rightarrow Fe_2O_3 + 6\,H^+ + 6\,e^- \quad (1)$$
$$2\,Cr + 3\,H_2O \rightarrow Cr_2O_3 + 6\,H^+ + 6\,e^- \quad (2)$$
$$Ni + H_2O \rightarrow NiO + 2\,H^+ + 2\,e^- \quad (3)$$
$$Mn + 2\,H_2O \rightarrow MnO_2 + 4\,H^+ + 2\,e^- \quad (4)$$
$$Mo + 2\,H_2O \rightarrow MoO_2 + 4\,H^+ + 4\,e^- \quad (5)$$
$$DE = 100\% \cdot \frac{Q_{diss}}{Q_{EC}} = 100\% \cdot \frac{\sum \frac{m_{Diss,Me}}{M_{Me}} \cdot z_{Me} F}{\int I dt} \quad (6)$$

With Q_{EC} as the transferred charge, Q_{diss} as the charge required for dissolution, $m_{Diss,Me}$, M_{Me}, and z_{Me} as the dissolved mass, molar mass, and transferred electrons for dissolution per element during the SFC-ICP-MS measurement, respectively, F as the Faraday constant, and I as current.

3 Microspectroscopy results

The change of reflectance on a freshly polished 316L sample was analyzed via microspectroscopy. The spot diameter of the employed 10x / NA 0.25 objective lens was 20 µm. To mitigate the influence of surface roughness induced by polishing scratches, the mean value of three-point measurements was considered. Reference measurements on Si were performed to obtain absolute reflectance values.

Since only a small variation in reflectance due to native oxide growth was expected, we evaluated the reflectance ($R(t)$) relative to the measurement on day 1 (R_0). The results are depicted in Figure S2 a for the spectral range from 440 – 950 nm. Here, no systematic change over time is observed. The acquired reflectance fluctuates with a maximum deviation of 0.5% compared to day 1. The small variations could be caused by surface inhomogeneities or simply represent the repetition accuracy of the setup.

To get an estimate of the expected reflectance change in the case of significant oxide growth, we modeled the reflectance of the 316L sample with varying thickness *d* of an assumed chromium oxide (Cr_2O_3) layer on top.[9] The modeling was performed using the transfer-matrix-method (TMM) approach introduced in our previous work.[10] Looking at the deviation in modeled reflectance relative to the bare 316L in Figure S2b, the expected difference for a Cr_2O_3 thickness of 1 nm for wavelengths below 600 nm already exceeds the measured deviation of 0.5%. In the case of thicker layers, a noticeably higher deviation is anticipated, reaching up to 5% at a thickness of 5 nm.

Based on these findings, no evidence for significant oxide growth was detected. Therefore, we assume that the majority of the self-limiting oxide formed prior to the first reflectance measurement. Measurements taken on the following days show no further growth beyond this point.

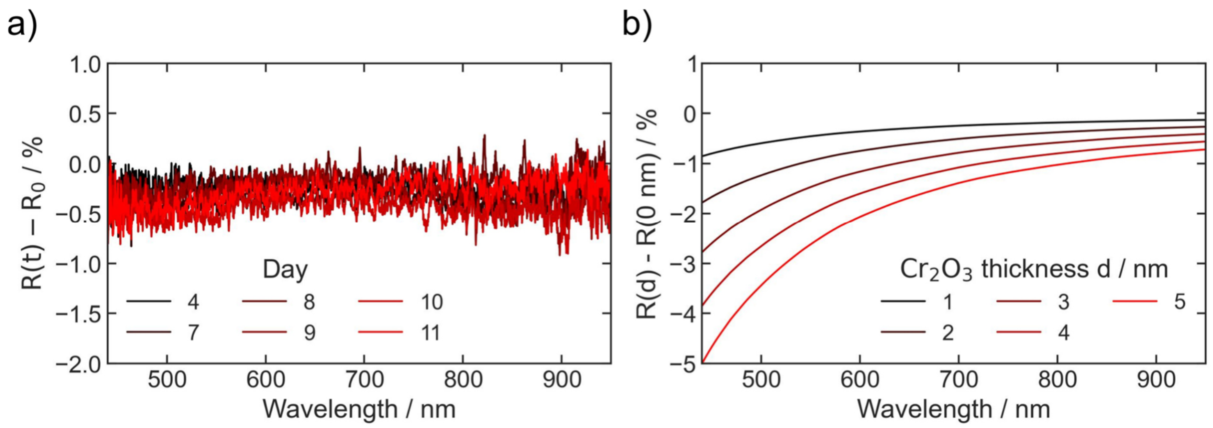

Figure S2. a) Difference in measured reflectance on the 316L sample over time relative to the measurement on Day 1. The results represent the mean value of three measurements. b) Difference in modeled reflectance for varying thicknesses of an assumed Cr_2O_3 thickness on top of the 316L sample relative to the bare sample.

4 SFC-ICP-MS

Table S2. Transferred charge during the SFC-ICP-MS measurement of 316L in 0.5 mM H_2SO_4 with different fluoride concentrations at T_{stage} = 60 °C displayed in Figure 1 in the manuscript (individual measurements refer to Figure S3). Average with standard deviation of three measurements.

F^- / ppm	$Q_{Hold\ initial}$ / mC	$Q_{1st\ cycle}$ / mC	$Q_{2nd\ cycle}$ / mC	$Q_{3rd\ cycle}$ / mC	$Q_{\mathrm{Hold\ final}}$ / mC	Q (total) / mC
0	0.23 ± 0.02	2.08 ± 0.02	1.59 ± 0.07	1.38 ± 0.07	0.017 ± 0.001	5.3 ± 0.2
1	0.21 ± 0.01	1.97 ± 0.05	1.54 ± 0.08	1.42 ± 0.17	0.031 ± 0.003	5.2 ± 0.3
5	0.26 ± 0.06	1.6 ± 0.08	1.29 ± 0.01	1.12 ± 0.08	0.082 ± 0.008	4.4 ± 0.2
20	0.65 ± 0.08	2.37 ± 0.15	1.98 ± 0.06	1.85 ± 0.09	0.51 ± 0.11	7.4 ± 0.1

Table S3. Maximum dissolution rates during the measurement of 316L in 0.5 mM H_2SO_4 with different fluoride concentrations at T_{stage} = 60 °C displayed in Figure 1 in the manuscript (individual measurements refer to Figure S3). Average with standard deviation of three measurements.

	$dM_{max}\ dt^{-1}\ dS^{-1}$ / ng s^{-1} cm^{-2}				
element	$Hold_{initial}$	1st cycle	2nd cycle	3rd cycle	$Hold_{final}$
0 ppm					
Fe	1.71 ± 0.1	1.82 ± 0.32	0.89 ± 0.17	0.75 ± 0.29	1.87 ± 0.23
Cr	0.27 ± 0.07	0.66 ± 0.06	0.33 ± 0.25	0.27 ± 0.2	0.66 ± 0.06
Ni	0.14 ± 0.03	0.29 ± 0.06	0.19 ± 0.13	0.16 ± 0.11	0.29 ± 0.06
Mn	0.032 ± 0.002	0.019 ± 0.005	0.017 ± 0.007	0.015 ± 0.006	0.032 ± 0.002
Mo	0.01 ± 0.003	0.02 ± 0.002	0.014 ± 0.004	0.013 ± 0.005	0.02 ± 0.002
1 ppm					
Fe	1.7 ± 0.09	2.17 ± 0.12	1.4 ± 0.2	1.7 ± 0.4	2.17 ± 0.12
Cr	0.15 ± 0.01	0.61 ± 0.04	0.36 ± 0.11	0.66 ± 0.45	0.78 ± 0.33
Ni	0.13 ± 0.1	0.22 ± 0.02	0.28 ± 0.13	0.28 ± 0.13	0.31 ± 0.1
Mn	0.024 ± 0.004	0.017 ± 0.002	0.025 ± 0.008	0.026 ± 0.008	0.03 ± 0.005
Mo	0.007 ± 0.001	0.019 ± 0.003	0.017 ± 0.003	0.029 ± 0.01	0.03 ± 0.009
5 ppm					
Fe	2.8 ± 0.98	4.36 ± 1.16	2.65 ± 0.16	2.43 ± 0.28	4.36 ± 1.16
Cr	0.37 ± 0.13	1.32 ± 0.47	0.83 ± 0.1	0.76 ± 0.16	1.34 ± 0.45
Ni	0.34 ± 0.23	0.57 ± 0.1	0.39 ± 0.04	0.32 ± 0.02	0.57 ± 0.1
Mn	0.032 ± 0.009	0.043 ± 0.004	0.04 ± 0.001	0.037 ± 0.005	0.043 ± 0.004
Mo	0.027 ± 0.013	0.053 ± 0.019	0.034 ± 0.002	0.035 ± 0.003	0.055 ± 0.017
20 ppm					
Fe	8.57 ± 0.51	15.45 ± 2.83	13.96 ± 2.71	13.65 ± 2.15	16 ± 2.7
Cr	1.36 ± 0.21	4.78 ± 1.02	3.71 ± 0.78	3.66 ± 0.45	4.78 ± 1.02
Ni	0.74 ± 0.16	2.31 ± 0.46	1.97 ± 0.31	1.92 ± 0.3	2.32 ± 0.46
Mn	0.105 ± 0.009	0.182 ± 0.041	0.187 ± 0.027	0.2 ± 0.022	0.215 ± 0.014
Mo	0.132 ± 0.025	0.234 ± 0.073	0.177 ± 0.055	0.166 ± 0.039	0.235 ± 0.07

Figure S3 displays the single SFC-ICP-MS measurements of 316L in 0.5 mM H_2SO_4 with different fluoride additions. As explained in the manuscript, differences in the dissolution profiles of 316L on different sample spots under the same measurement conditions occur with potential cycling, which is more pronounced with increased fluoride concentration. This effect is mainly caused by unexpected dissolution increases that differ per measurement and are often accompanied by transients in the current signal. Exemplary, this can be observed for 0 ppm fluoride for spot 2 around 885 s and 1160 s. For 5 ppm fluoride, it can be observed for spot 2 around 160 s and for 20 ppm at spot 1 around 1190 s. However, for the latter one, a current increase over a longer time, and not only a transient is observed. Still, not every increase in dissolution appears simultaneously as an increase in the dissolution rate and vice versa. For example, for spot 2 at 1 ppm fluoride, an increase in dissolution rate, especially of Ni and Mn, is observed while only a slight noise in the current density signal is apparent. On the other hand, for spot 2 of 5 ppm fluoride, a transient occurs around 360 s, but no unexpected increase in the dissolution rate is observed. As mentioned in the manuscript, we speculate that these phenomena could be caused by a peel-off of the formed oxide layer due to the electrolyte flow or pitting [11-12] As fluoride is known to induce pitting on 316L[13] and later proven by SEM images and laser profilometry for our measurements, we assume that pitting corrosion being the most likely process. While pitting might only be metastable for low fluoride concentrations, its severity increases with higher concentrations, as also indicated by the SEM images. The not directly relatable observation of unexpected dissolution and current transient could be explained by overlapping phenomena that “cover” the other.

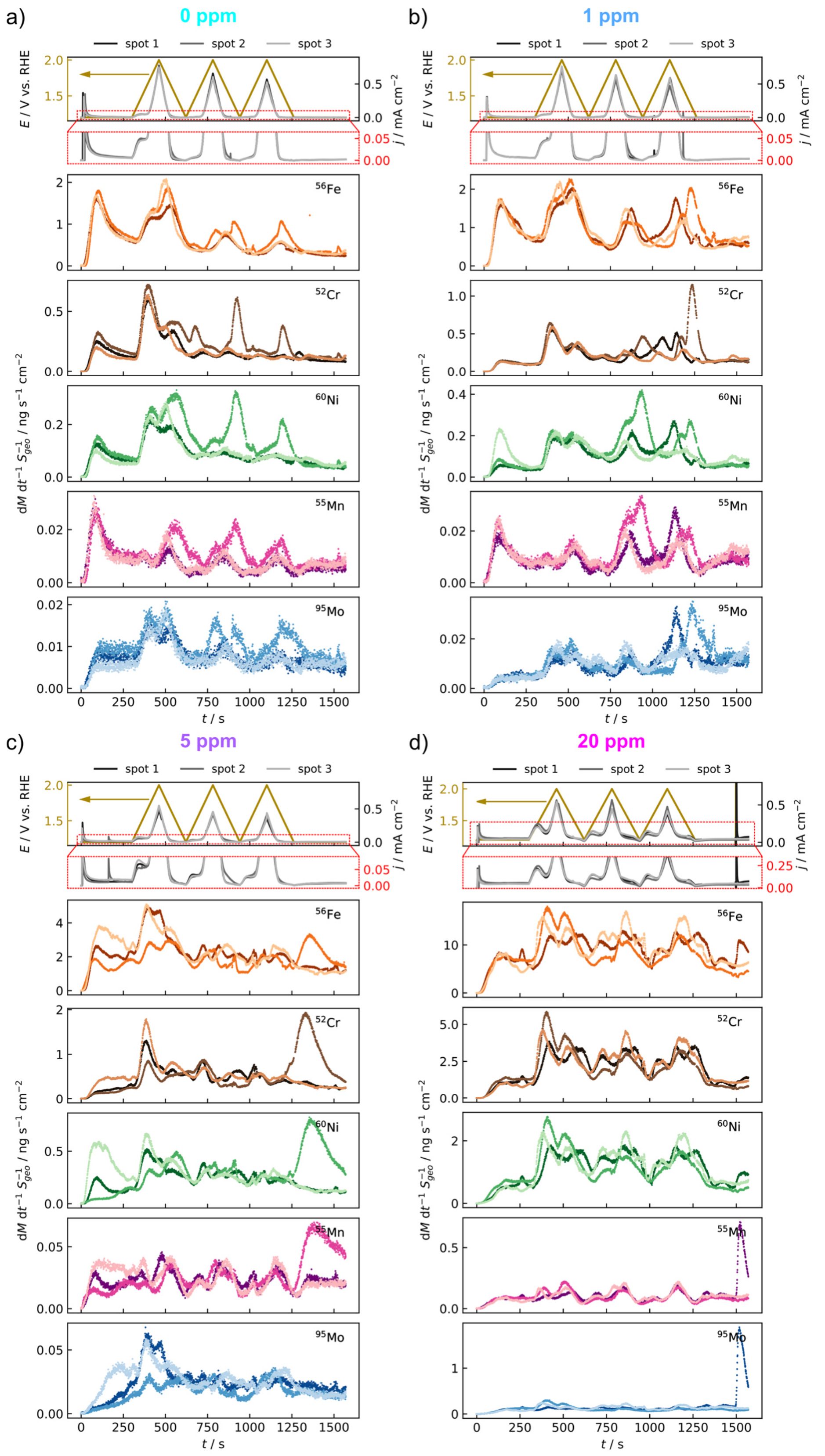

Figure S3. Individual SFC-ICP-MS measurements of 316L in 0.5 mM H_2SO_4 at T_{stage} = 60 °C with different fluoride additions: a) 0 ppm, b) 1 ppm, c) 5 ppm, and d) 20 ppm. From top to bottom: Potential profile (beige) and current density, as well as dissolution rates of Fe, Cr, Ni, Mn, and Mo. Three measurements per fluoride concentration on three independent sample locations (dark to light). The averages with standard deviation per fluoride concentration of the displayed measurements are shown in Figure 1 in the manuscript.

For the measurement at spot 1 of 316L in 0.5 mM H_2SO_4 with addition of 20 ppm fluoride, a very severe spike in the potential and current signal is observed during the final 1.2 V hold which results in multiple overloads of the

potentiostat in the following section until both, applied potential and current measured, are stable again. At the same time, there is a drastic increase in the dissolution rates of all elements, but mainly of Mo and Mn (Figure S3d). To exclude the influence of a bubble, we checked the raw signal during the ICP-MS measurements of the analytes and internal standard (Figure S4). If this phenomenon would be artificially caused by a bubble (e.g., due to oxygen evolution reaction or air trapped in the inlet SFC tubing) touching the WE surface, we would expect to see that by fluctuation in the ICP-MS signal of the internal standard (for example, as visible around 1000 s). As this is not the case, we expect that this phenomenon is caused by the measurement itself, such as a breakthrough of the oxide layer or severe pitting corrosion.

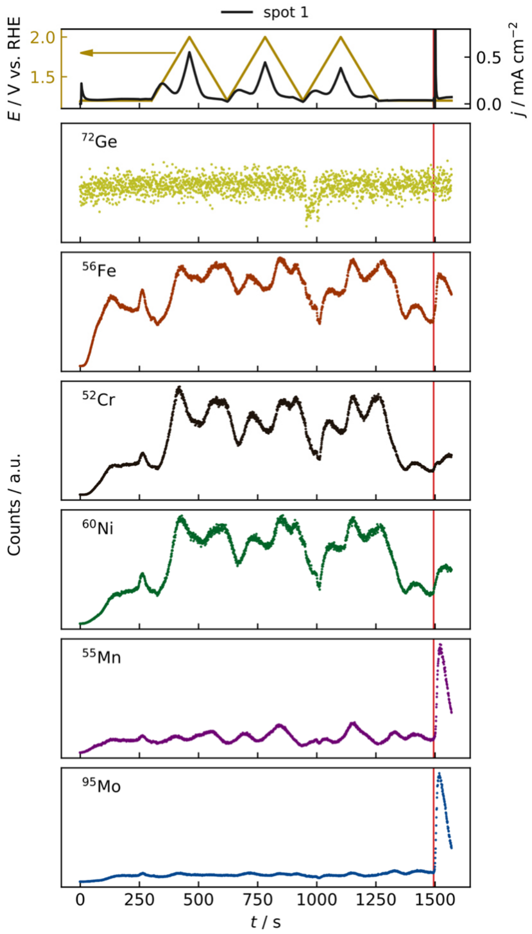

Figure S4. SFC-ICP-MS measurements of 316L in 0.5 mM H_2SO_4 + 20 ppm fluoride – spot 1 from Figure S4d. From top to bottom: Potential profile (beige) and current density, raw counts of Ge (internal standard) and Fe, Cr, Ni, Mn, and Mo (analytes).

Figure S5 shows the DE for different regions during the SFC-ICP-MS measurements. It becomes apparent that the DE for the measurement with 20 ppm fluoride is constantly high whereas for the measurements with 0, 1, and 5 ppm fluoride, the DE is higher during the first current increase (refers to the regime of the first current increase per cycle before the second sharp current increase) and lower for the rest of the cycle (called 2nd peak). Of course, this analysis is slightly influenced by the sudden, unexpected dissolution occurring during the measurements. However, it shows that at a high fluoride concentration, the DE is always high and dominating, while at lower concentrations, the DE is high at low potentials, but other reactions, such as oxygen evolution or oxide layer formation, take place at a higher rate at elevated potentials and the backward scan.

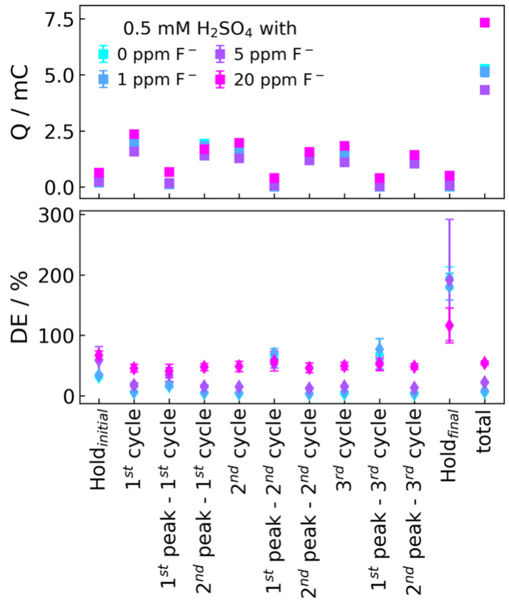

Figure S5. Transferred charge *Q* and dissolution efficiency DE during the SFC-ICP-MS measurements of 316L in 0.5 mM H_2SO_4 with different fluoride concentrations. The 1st peak per cycle refers to the region with a stable current/evolved peak in current density, while the 2nd peak is the regime from the sharp current increase until the end of the cycle.

5 Microscopic investigation

5.1 Pseudo-identical location scanning electron microscopy

We investigated the area of measurements on the sample before and after measurement with pseudo-identical location SEM to assign any observed changes to corrosion phenomena and not to structural effects that were apparent on the sample beforehand. As the sample area is rather large and the corrosion phenomena are not homogenously distributed over the sample, images with higher magnification before and after measurement are not taken at the exact same position inside the measurement spot; therefore, we call it pseudo-identical location. All pristine SEM images show a similar structure – scratches are visible due to the final polishing of the sample. Moreover, small black and white specs can be seen as highlighted exemplary in Figure S7a-i. These could be oxide or sulfide inclusions, which are often possible initiation sites of pitting.[14-15]

Figure S6a displays low-magnification SEM images of the spot after measurement and of an exemplary pristine area. Figure S6b puts the observed corroded area in Figure S6a in relation to the used O-ring as a sealant. It becomes apparent that the corroded area is larger than the inner diameter of the O-ring due to the cross-section diameter of the O-ring, which exposes an additional area underneath the O-ring. As the O-ring is pressed with a constant force controlled by a force sensor attached to the SFC, a constant compression of the O-ring and thereby active area will achieved. Still, the reported dissolution rates, current density, and the calculation of the dissolved ML are based on the inner diameter of the O-ring.

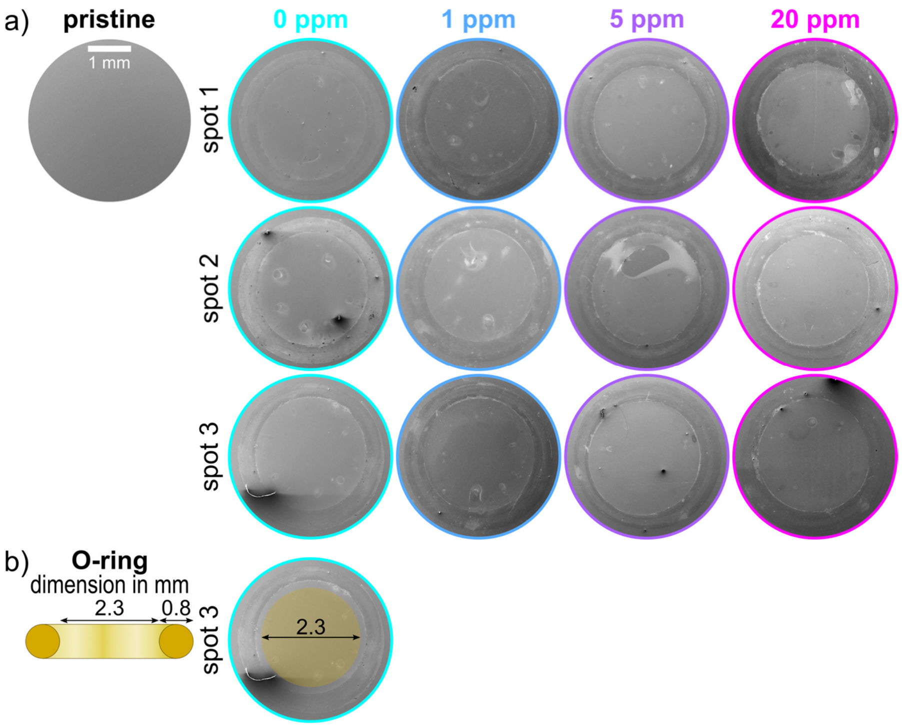

Figure S6. a) Low magnification SEM images of all SFC-ICP-MS spots after measurement and one exemplary pristine area. b) dimension of the used O-ring to seal the samples' active area and SEM image with its indicated inner diameter in the area with visible corrosion of spot 3 after SFC-ICP-MS with 1 ppm fluoride. The scale bar refers to all SEM images. Brightness and contrast of SEM images were edited to enhance visibility

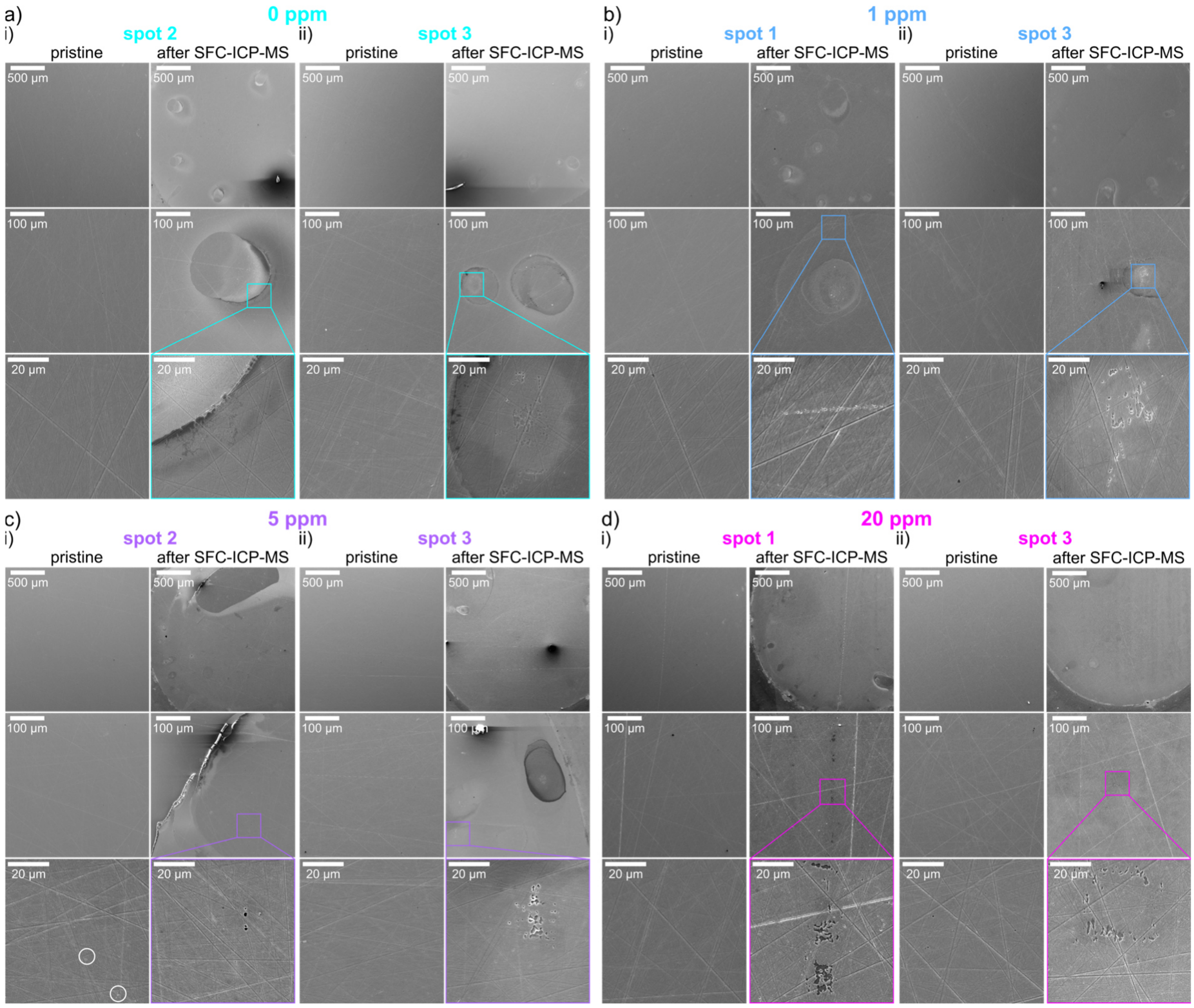

Figure S7. SEM images before and after SFC-ICP-MS measurements of 316L in 0.5 mM H_2SO_4 with different fluoride concentrations at T_{stage} = 60 °C displayed in Figure 1 in the manuscript (individual measurements refer to Figure S3). All images were contrast-enhanced to improve visibility.

5.2 Laser profilometry

Figure S8a displays additional laser profilometry measurements of 316L after SFC-ICP-MS measurements. The scratches are caused by the final polishing of the sample and are also apparent on the pristine sample. As mentioned in the manuscript, these laser profilometry measurements hint towards deposits on the sample with a recess in the middle. However, not for every deposit a recess is measured (e.g., 5 ppm right measurement). SEM images of the suspected areas after 20 ppm (Figure S8b) also show structural changes at the suspected areas.

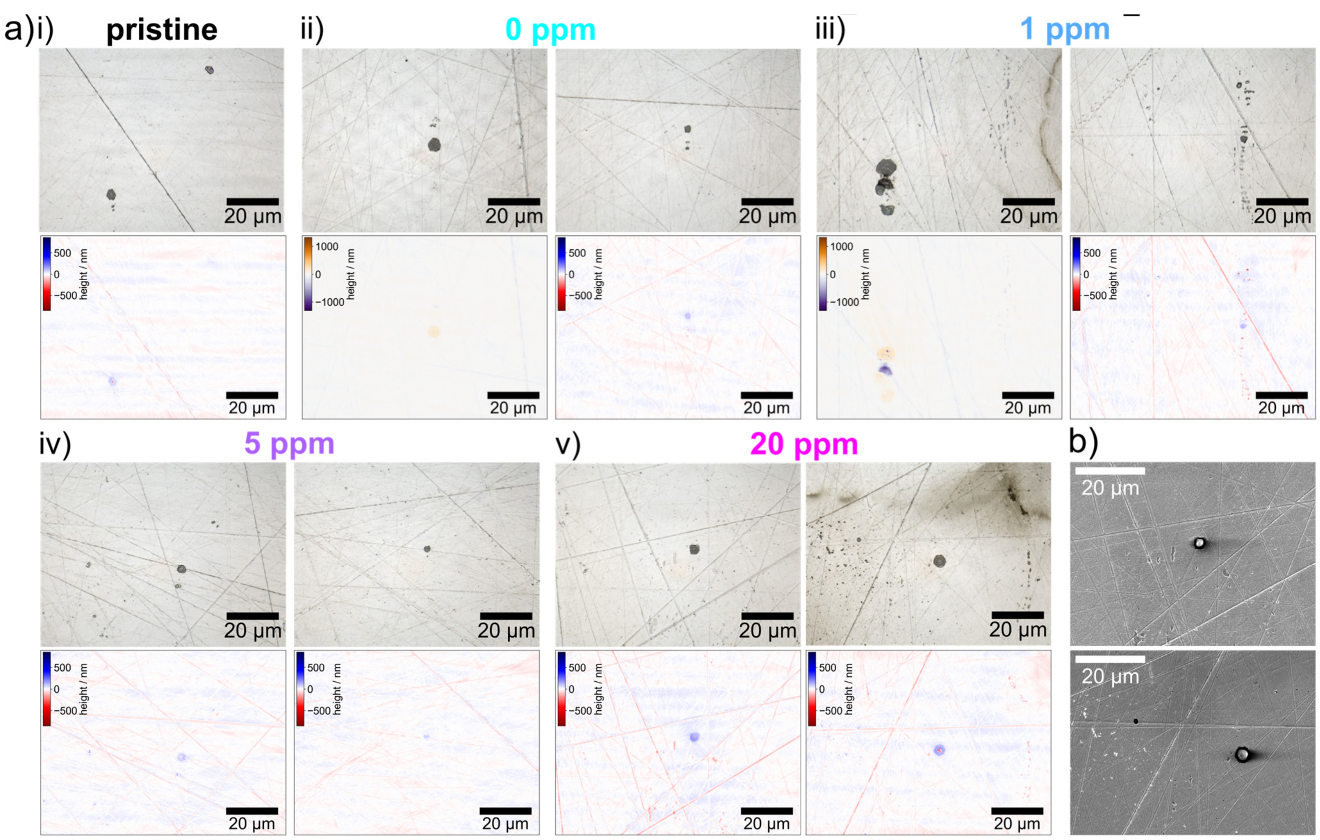

Figure S8. a) Laser optical micrograph and corresponding laser profilometry height map of a pristine sample (i) and after SFC-ICP-MS measurement with different fluoride concentrations (ii – 0 ppm, iii – 1 ppm, iv – 5 ppm, and v – 20 ppm). b) SEM images of the measurement areas shown in a-v.ad. SEM images were edited to enhance visibility.

5.3 STEM-EDXS of FIB lamellae

We prepared a FIB-lift out of a pristine area (similar position as indicated in Figure 3a, pristine) as well as at one of the deposits with apparent recess by laser profilometry measurements (position marked in Figure 3a, 20 ppm) and investigated the nanostructure by scanning transmission electron microscopy with energy-dispersive X-ray spectroscopy (STEM-EDXS) (Figure S9). The pristine area (Figure S9a) shows the homogeneous composition of the sample's bulk, comparable to the 316L investigated in our previous study and theoretical composition (Table S1).[4] After measurement at 20 ppm fluoride, the FIB lamella analysis reveals that the suspicious area is an inclusion with a depth of approximately 2.3 µm and a width of 4.6 µm. The inclusion mainly consists of manganese, aluminum, and magnesium (Table S4-Area1) with a high oxygen content. The EDX detection of sulfur was not possible due to the overlap with the Mo signal. The detection of fluor was not possible due to the overlap of the of F K_α peak with the Fe L_α peak.[16] The STEM-EDXS analysis (Figure S9b-ii) of a structural anomaly inside the inclusion (highlighted with a green square in Figure S9b-i) shows that this is caused by additional silicon and calcium oxide inclusions (composition Table S4-Area2). The anomaly has a width of ~550 nm and a depth of ~250 nm. The indicated protruding height of the inclusion of ~100 – 150 nm by laser profilometry is in good agreement with the result of the STEM-EDXS analysis of the FIB-lamella. Such inclusions are generally known as pit initiation sites in stainless steel.[17-19] Even though a recess is indicated by the laser profilometry measurements across the inclusion with a depth of ~800 nm and size of ~1 µm, this was not verifiable by the prepared FIB-lamella. This could have two reasons. First, it is possible that the lift-out was not directly above the apparent pit, as this is only a smaller area of the inclusion. Second, and more likely, it is an artifact measured in the laser profilometry measurements due to a lens effect of the inclusion (most probably a manganese aluminum oxide that is partially transparent for the laser beam in contrast to the metal substrate). For example, the laser profilometry measurement of such an inclusion on a pristine sample (Figure S8-i) also appears with a recess in the middle. Therefore, stainless steel's exact IL-location analysis could give valuable insights into localized corrosion phenomena to assign structural anomalies to corrosion and ensure they were not already apparent on the sample beforehand.

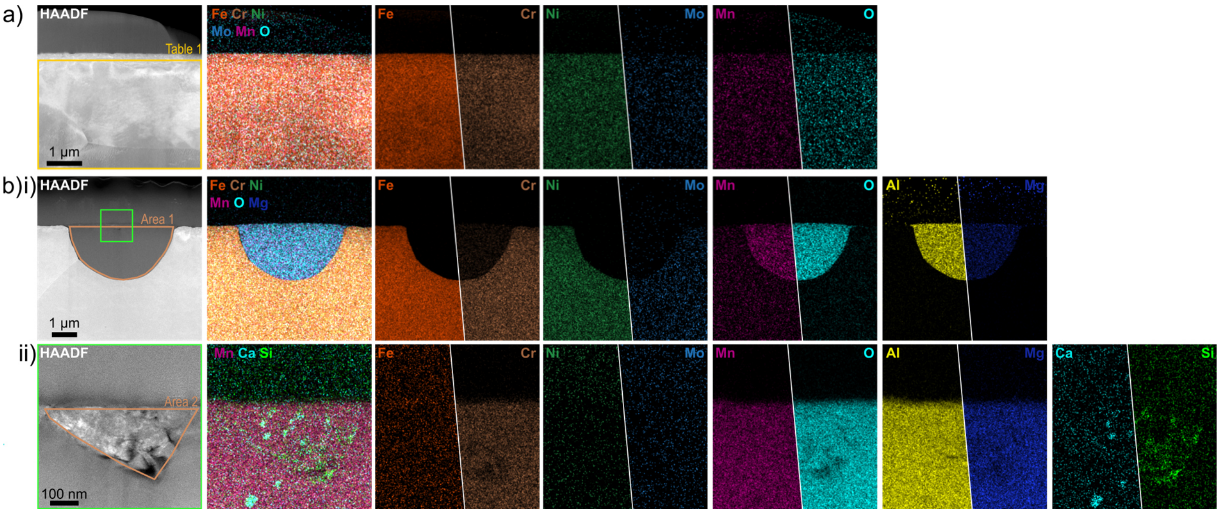

Figure S9. STEM-EDXS analysis of the FIB lamellae. HAADF scans with corresponding EDX spectrum images for (a) the pristine area and (b) after measurement with 20 ppm fluoride. The integrated spectrum averages of the area in the yellow rectangle in a) was used for verification of the pristine 316L composition given in Table S1, whereas compositions of regions indicated as Area 1 and 2 in b) are given in Table S4.

Table S4. Composition of the inclusion in the sample in wt%. Integrated spectrum averages of the area indicated in Figure S9b. Please refer to Table S1 for 316L's nominal composition.

	Fe	Cr	Ni	Mo	Mn	O	Al	Mg	Ca	Si
Area 1	0.5 ± 0.1	9.3 ± 1.3	0.1 ± 0.1	1.6 ± 0.3	15 ± 2.2	37.1 ± 2.4	27.9 ± 4.1	8.3 ± 1.7		
Area 2	0.5 ± 0.1	9.3 ± 1.3	0.04 ± 0.01	1.4 ± 0.2	13.5 ± 2	37.9 ± 2.4	28 ± 4.1	7.9 ± 1.6	0.2 ± 0.1	0.4 ± 0.1

6 Calculation of specific feed water flow rate

To estimate the accumulation of fluoride ions on single cell level, fluoride emission rates (FER) reported in literature[20-21] were used to calculate the critical specific feed water flow rate to accumulate 1 ppm fluoride in the electrolyzer cell. The calculation is exemplary shown in equation (7).

$$critical\ specific\ feed\ water\ flow\ [mL\ \min^{-1}\ cm^{-2}] = \frac{FER}{1\ ppm} = \frac{3.5\ \mu g\ L}{1\ mg\ h\ cm^2} \cdot \frac{1}{60} = 0.06 \tag{7}$$

References

[1] E. Kuhnert, V. Hacker, M. Bodner, P. Subramanian, "A Review of Accelerated Stress Tests for Enhancing MEA Durability in PEM Water Electrolysis Cells" *Int. J. Energy Res.* **2023**, *2023*, 1-23.

[2] P. Fruhwirt, A. Kregar, J. T. Torring, T. Katrasnik, G. Gescheidt, "Holistic approach to chemical degradation of Nafion membranes in fuel cells: modelling and predictions" *Phys. Chem. Chem. Phys.* **2020**, *22*, 5647-5666.

[3] L. Fiedler, T. C. Ma, B. Fritsch, J. H. Risse, M. Lechner, D. Dworschak, M. Merklein, K. J. J. Mayrhofer, A. Hutzler, "Stability of Bipolar Plate Materials for Proton-Exchange Membrane Water Electrolyzers: Dissolution of Titanium and Stainless Steel in DI Water and Highly Diluted Acid" *ChemElectroChem* **2023**, *10*, e202300373.

[4] L. Fiedler, T.-C. Ma, B. Fritsch, M. Dierner, D. Hoffmeister, C. Rubach, J. Will, T. Przybilla, E. Spiecker, D. Dworschak, K. J. J. Mayrhofer, A. Hutzler, "Stainless Steel 316L as Bipolar Plate Material in Proton Exchange Membrane Water Electrolyzer: The Influence of Potential and Temperature on Dissolution Stability" *Mater. Today Sustain.* **2025**, 101155.

[5] J. C. Stinville, P. Villechaise, C. Templier, J. P. Riviere, M. Drouet, "Plasma nitriding of 316L austenitic stainless steel: Experimental investigation of fatigue life and surface evolution" *Surf. Coat. Technol.* **2010**, *204*, 1947-1951.

[6] F. Borgioli, A. Fossati, E. Galvanetto, T. Bacci, G. Pradelli, "Glow discharge nitriding of AISI 316L austenitic stainless steel: Influence of treatment pressure" *Surf. Coat. Technol.* **2006**, *200*, 5505-5513.

[7] AZO Materials, "*Stainless Steel - Grade 316L - Properties, Fabrication and Applications (UNS S31603)*", can be found under https://www.azom.com/article.aspx?ArticleID=2382, (accessed: (15.11.2024)).

[8] D. Göhl, H. Rueß, A. M. Mingers, K. J. J. Mayrhofer, J. M. Schneider, M. Ledendecker, "Electrochemical Passivation Properties of Valve Transition Metal Carbides" *J. Electrochem. Soc.* **2022**, *169*, 011502.

[9] Z. Wang, F. Di-Franco, A. Seyeux, S. Zanna, V. Maurice, P. Marcus, "Passivation-Induced Physicochemical Alterations of the Native Surface Oxide Film on 316L Austenitic Stainless Steel" *J. Electrochem. Soc.* **2019**, *166*, C3376-C3388.

[10] J. Schwarz, M. Niebauer, M. Kolesnik-Gray, M. Szabo, L. Baier, P. Chava, A. Erbe, V. Krstic, M. Rommel, A. Hutzler, "Correlating Optical Microspectroscopy with 4x4 Transfer Matrix Modeling for Characterizing Birefringent Van der Waals Materials" *Small Methods* **2023**, *7*, e2300618.

[11] J. Soltis, "Passivity breakdown, pit initiation and propagation of pits in metallic materials – Review" *Corros. Sci.* **2015**, *90*, 5-22.

[12] E. McCafferty, "Crevice Corrosion and Pitting" in *Introduction to Corrosion Science* (Ed.: E. McCafferty), Springer New York, New York, NY, **2010**, pp. 263-313.

[13] Y. Yang, L.-j. Guo, H. Liu, "Effect of fluoride ions on corrosion behavior of SS316L in simulated proton exchange membrane fuel cell (PEMFC) cathode environments" *J. Power Sources* **2010**, *195*, 5651-5659.

[14] J. Stewart, D. E. Williams, "The initiation of pitting corrosion on austenitic stainless steel: on the role and importance of sulphide inclusions" *Corros. Sci.* **1992**, *33*, 457-474.

[15] V. Vignal, C. Voltz, S. Thiébaut, M. Demésy, O. Heintz, S. Guerraz, "Pitting Corrosion of Type 316L Stainless Steel Elaborated by the Selective Laser Melting Method: Influence of Microstructure" *J. Mater. Eng. Perform.* **2021**, *30*, 5050-5058.

[16] G. G. Williams, "1. X-Ray properties of the elements - 1.1 Electron binding energies" in *X-Ray Data Booklet, Vol. 3* (Ed.: A. C. Thompson), Lawrence Berkeley National Laboratory, Berkeley, **2009**, pp. 1-1 - 1-7.

[17] N. Hara, K. Hirabayashi, Y. Sugawara, I. Muto, "Improvement of Pitting Corrosion Resistance of Type 316L Stainless Steel by Potentiostatic Removal of Surface MnS Inclusions" *International Journal of Corrosion* **2012**, *2012*, 1-6.

[18] Z. Duan, C. Man, H. Cui, Z. Cui, X. Wang, "Formation mechanism of MnS inclusion during heat treatments and its influence on the pitting behavior of 316L stainless steel fabricated by laser powder bed fusion" *Corrosion Communications* **2022**, *7*, 12-22.

[19] Z. Zhang, Z. Zhao, X. Li, L. Wang, B. Liu, P. Bai, "Effect of heat treatments on metastable pitting of 316L stainless steel fabricated by selective laser melting" *J. Mater. Res. Technol.* **2022**, *21*, 1903-1914.

[20] E. Kuhnert, M. Heidinger, A. Bernroitner, Ö. Kiziltan, E. Berger, V. Hacker, M. Bodner, "Fluoride emission rate analysis in proton exchange membrane water electrolyzer cells" *Front. Energy Res.* **2024**, *12*.

[21] S. H. Frensch, F. Fouda-Onana, G. Serre, D. Thoby, S. S. Araya, S. K. Kær, "Influence of the operation mode on PEM water electrolysis degradation" *Int. J. Hydrogen Energy* **2019**, *44*, 29889-29898.
